# A Microfluidic Prototype for High-Frequency, Large Strain Oscillatory Flow Rheometry

**DOI:** 10.3390/mi13020256

**Published:** 2022-02-03

**Authors:** Alfredo Lanzaro, Xue-Feng Yuan

**Affiliations:** Institute for Systems Rheology, Guangzhou University, No. 230 West Outer Ring Road, Higher Education Mega-Center, Panyu District, Guangzhou 510006, China; xuefeng.yuan@gzhu.edu.cn

**Keywords:** microfluidic rheometry, high frequency characterisation, LAOS

## Abstract

We introduce a “Rheo-chip” prototypical rheometer which is able to characterise model fluids under oscillatory flow at frequencies *f* up to 80 Hz and nominal strain up to 350, with sample consumption of less than 1 mL, and with minimum inertial effects. Experiments carried out with deionized (DI) water demonstrate that the amplitude of the measured pressure drop $\Delta P_{M}$ falls below the Newtonian prediction at f≥ 3 Hz. By introducing a simple model which assumes a linear dependence between the back force and the dead volume within the fluid chambers, the frequency response of both $\Delta P_{M}$ and of the phase delay could be modeled more efficiently. Such effects need to be taken into account when using this type of technology for characterising the frequency response of non-Newtonian fluids.

## 1. Introduction

Characterising the mechanical response of low-viscosity (μ = $10^{-3}$–0.1 Pa·s) complex fluids at high frequencies is of great utility for understanding a wide number of industrial processes. In drop-on-demand inkjet printing, droplets are generated or deposited at frequencies *f* on the order of KHz [1]. The presence of high molecular weight polymer additives in the dilute or semi-dilute unentangled regime results in a longest relaxation time λ on the order of 0.1–1 ms [2,3,4,5,6], such that the characteristic Deborah number of the inkjet process is De = λω≤ 25, where ω=2πf. Over such range of *f*, the viscoelasticity of the polymer solution has a profound impact on the printing process because it suppresses the formation of small satellite droplets, and causes the onset of long-lived filaments [7]. Understanding the high-frequency behaviour of complex fluids is also important for the biopharmaceutical industry. Dense solutions of proteins of medical use, such as monoclonal antibodies, create sample-spanning networks under attractive conditions, which in turn give rise to nonlinear rheological behaviour [8,9,10,11]. The elastic modulus of such protein solutions measured at frequencies on the order of kHz has been demonstrated to correlate well with the strength of the protein–protein attractive interactions [12,13,14].

Due to inertial effects, conventional rotational rheometers can characterise the viscoelastic response of low-viscosity fluids only in the limit of low frequencies. For example, in the case of a cone and plate or plate-plate geometry with radius $R_{1}$ = 30 mm, the Reynolds number $Re=\gamma \omega R_{1}^{2}/\nu$ where ν = 10${}^{-6}$–$10^{-4}$ m${}^{2}$/s and γ≈ 0.01, is on the order of unity for *f* on the order of a few Hz. To overcome this problem, the Piezoelastic Axial Vibrator (PAV) has been developed [15]. By applying a squeeze flow to the tested sample, the PAV is able to produce a viscoelastic characterisation of complex fluids up to $10^{3}$ Hz, while the required sample volume is ≈100 μL. However, the PAV also involves the presence of a fluid/air interface, which is disadvantageous as it might induce the formation of aggregates if amphiphilic molecules (i.e., proteins) are dissolved in the sample fluid. From this viewpoint, the use of microfluidic channels with plasma-treated internal surfaces eliminates the presence of an open interface, while the amount of required fluid can in principle be reduced up to a few nanolitres.

Additionally, microfluidics offer the possibility of studying the viscoelastic behaviour of low-viscosity complex fluids over a range of frequencies approximately one order of magnitude higher than what is achievable by rotational rheometers. For oscillatory flows through straight ducts and with zero mean speed, the relative importance of inertial effects with respect to viscous forces is quantified by the Womersley number [16], $Wo=D_{H}\sqrt{\omega /\nu }$. For Wo>>1, inertial effects dominate the flow. In the case of microfluidic channels, where $D_{H}$ is on the order of 100 μm, Wo is low or moderate up to *f* = 100 Hz, approximately [17].

Another advantage is represented by the maximum amplitude of the imposed strain. Most of the solutions previously adopted in the literature to generate an oscillatory flow rely on the use of piezo actuators which pump fluids through a reservoir by means of a membrane, and then through the microchannel. Since the membrane radius is usually *R*≈ 5 mm, a strain amplitude $\gamma =R^{2}d_{M}/D_{H}^{3}\approx 10^{3}$ for a membrane displacement amplitude $d_{M}$ = 50 μm can in theory be achieved. Because these values of γ are well above the maximum obtainable by conventional rotational rheometers, we see that microfluidics offer allow studying low-viscosity fluids under Large Amplitude Oscillatory Strain (LAOS) flows over an essentially unexplored region of the f−γ diagram [18].

In the recent years, several authors have studied the high frequency behavour of microfluidic devices obtained by combining piezo actuators and membranes with straight channels [17], fluid chambers [19] or cross-slot flow geometries [20]. At the best of our knowledge, methods specifically aimed at studying high-frequency LAOS of complex fluids have not yet been proposed. In this work, we present a microfluidic prototype which aims at characterising low-viscosity complex fluids under oscillatory shear flow over a range of *f* = 0.05–80 Hz, and nominal strain 20 ≤γ≤350. The technology is hereby validated using a Newtonian fluid, in order to evaluate the optimum window of *f* and γ that could be used for rheometric purposes.

## 2. “Rheo-Chip” Setup for Oscillatory Flow Measurements in Microfluidics

**Figure 1:**
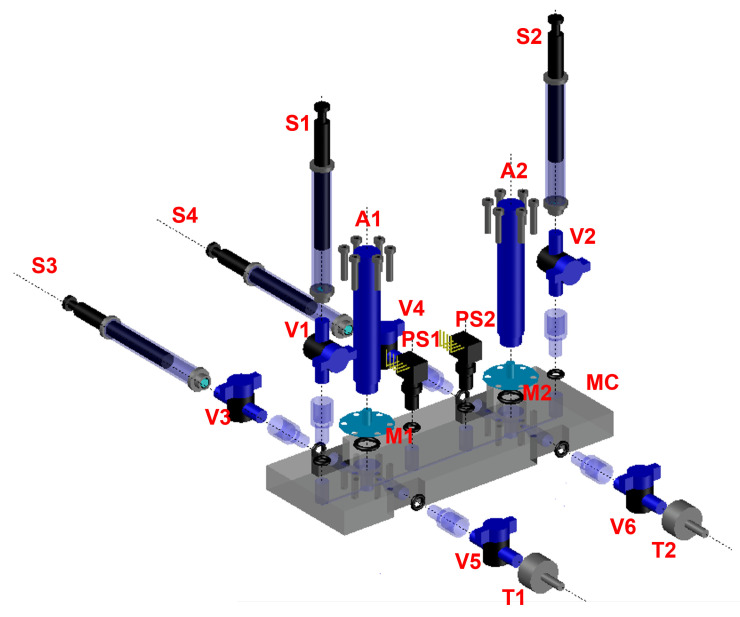
A 3D model of the Rheo-chip prototype for oscillatory flow. The microfluidic chip, labeled as MC in the Figure, is connected to the two piezo actuators A1 and A2 by means of the membranes M1 and M2. Before the experiments, the microchip is flipped by 90 degrees, and the test fluid is loaded from a container into the fluid chambers 1 and 2 by means of the metallic tips T1 and T2 and the syringes S3 and S4. After that, the valves V3, V4, V5 and V6 are closed. To perform the experiments, the syringe S1 is also loaded with the test fluid, and then the fluid is pushed through the microchannel. Before the experiment starts, the valves V1 and V2 are also closed. During the experiments, the PS1 and PS2 sensors measure the $P_{0}$ and $P_{1}$ gauge pressures respectively, so that ΔP = $P_{0}-P_{1}$ is obtained.

**Figure 2:**
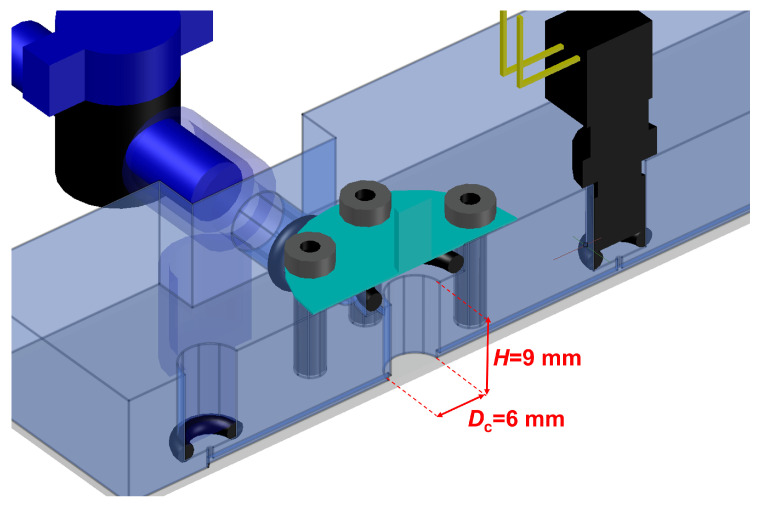
The details of one of the two fluid chambers.

In this work, we use a prototype based on the “Rheo-chip” technology [21,22] specifically developed for performing rheometric measurements in oscillatory flow. The core part of the device is a polymethil metaacrylate (PMMA) microfluidic chip made by standard soft lithography methods (Epigem LTD, Redcar, UK). A schematic detail of the device is given in Figure 1 and Figure 2. The microchip features a straight channel with a rectangular cross section (*w* = 800 μm, *h* = 50 μm) along which two pressure sensors PS1 and PS2 (range 0–30 psi, from Honeywell, Charlotte, NC, USA) are placed to read the pressure drop ΔP = $P_{0}-P_{1}$ along a distance *L* = 30 mm. The chip also comprises two fluid chambers, which serve for hosting two thin aluminum membranes (thickness = 0.25 mm) which are used to drive the motion of the sample. Each one of the two fluid chambers comprises sidewise extra inlets and outlets which are used to completely fill the chambers with sample before the experiments are run (see the caption of Figure 1). Both the pressure sensors were located at a distance $L_{P}$ = 25 mm from the fluid chambers, which excludes entry effects on the measured ΔP ($L_{P}/h>>$1). The total sample volume, obtained by summing the volume of the two chambers with that of the microfluidic channel, is approximately 0.5 mL. The microchips were treated with oxygen plasma to make the internal surface hydrophilic. The aluminum membranes were connected to two PI 841.30 piezoelectric actuators (Physik Instrumente GmbH, Eschbach, Germany) equipped with strain gauges for measuring the actuator displacement. The actuators have a travel range = 52 μm, a resonant frequency = 10 kHz with no load, and a resolution of the displacement of approximately 5.2 μm per applied volt. The piezo actuators were coupled with a PI E-500 10:1 voltage amplifier. The amplifier, the strain gauges from the piezos and the pressure sensors were then connected to a NI-cRIO controller (National Instruments, Austin, TX, USA). It generates signals with a maximum amplitude of 1 V and a sampling rate much larger than the frequency, and acquires the actuators’ displacement and the pressure drop signals. The voltage signals sent from the controller to the amplifier are two sine waves in anti-phase with each other, so that the corresponding displacements of the A1 and A2 actuators can be written as $d_{1}\left(t\right)$ = $d_{M}\sin\left(\omega t\right)$ and $d_{2}\left(t\right)$ = $d_{M}\sin\left(\omega t+\pi \right)$, that is, the two actuators worked in a “push-and-pull” modality.

**Figure 3:**
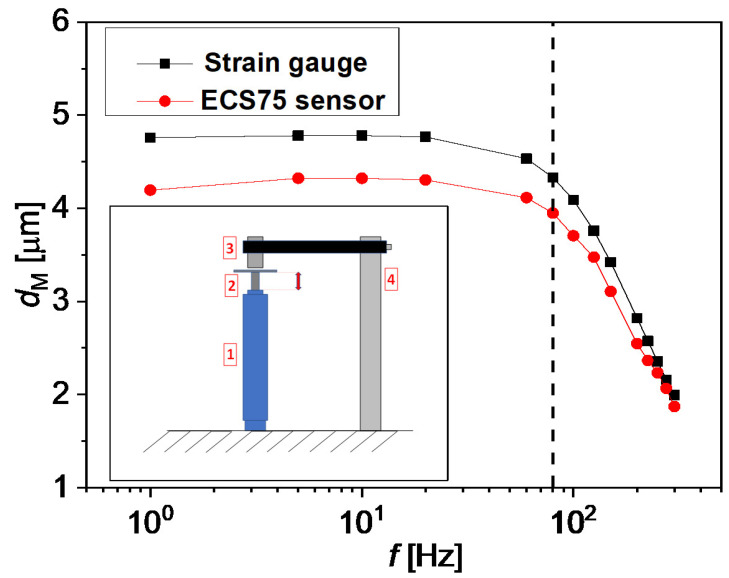
The amplitude of displacement of the A1 actuator as measured by the strain gauge (black) and by the ECS75 sensor (red) for an applied voltage of 0.1 V is plotted as a function of *f*. In the inset, the experimental setup of the ECS75 coupled with the piezo is shown: here, 1 is the actuator, 2 is the membrane, 3 is the ECS75 sensor, and 4 is the sensor holder.

Before connecting the actuators to the microfluidic chips, it is important to test their performance when no back force is applied. In Figure 3, the $d_{M}$ of the A1 actuator at a fixed amplitude of the applied voltage (0.1 V), is plotted versus *f*. The measurements were performed with both the strain gauge and a PI ECS75 capacitive sensor, and a close agreement between the two techniques was found. $d_{M}$ is linear versus *f* up to 80 Hz ca., and then it quickly decays with *f* for larger values of the imposed frequency. All the experiments presented in this work were carried out with DI water at 25 ${}^{\circ }$C (μ = 10${}^{-3}$ Pa·s, ρ = 10${}^{3}$ Kg/m${}^{3}$, ν=μ/ρ = 10${}^{-6}$ m${}^{2}$/s).

## 3. Results and Discussion

### 3.1. Strain Dependence of Pressure Drop at Low Frequency

**Figure 4:**
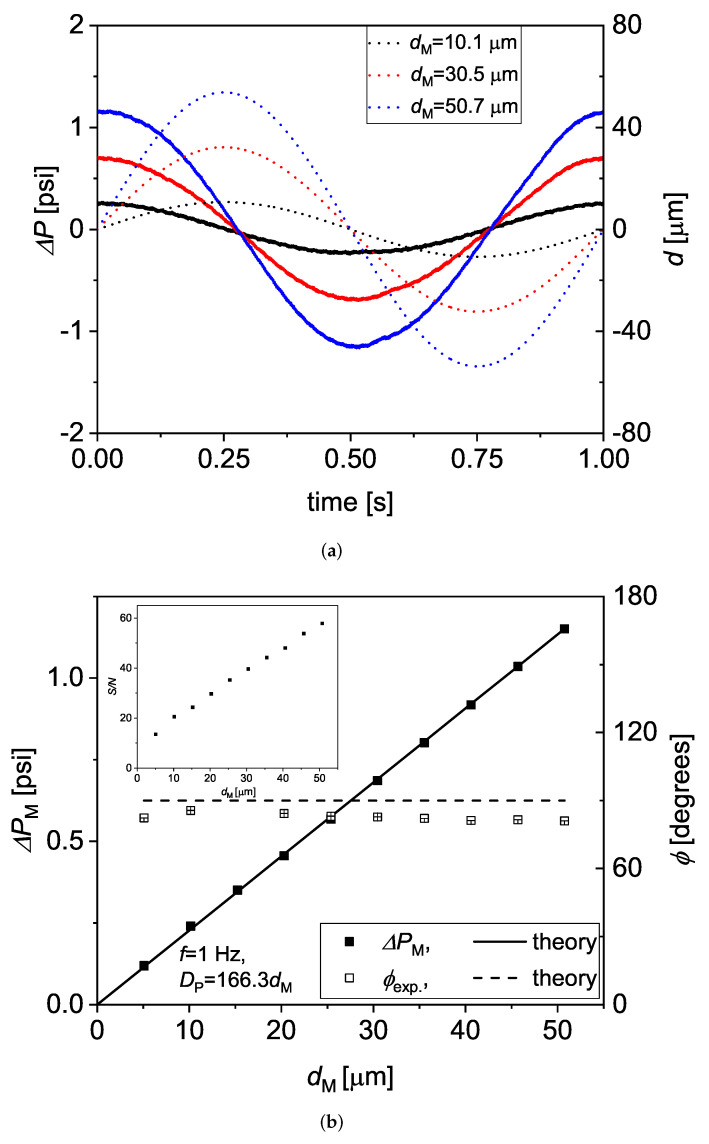
(**a**) the time dependence of the measured pressure drop $\Delta P\left(t\right)$ (continuous lines) for three different sinusoidal displacement curves $d_{1}\left(t\right)$ of the A1 actuator (the displacement amplitude $d_{M}$ is given in the legend). (**b**) the amplitude of the measured pressure drop, $\Delta P_{M}$, and the phase delay, $\phi_{\exp.}$, plotted as a function of $d_{M}$ together with the Newtonian prediction for rectangular ducts (see Equations (1)–(3)). The $\Delta P_{M}$ and $\phi_{\exp.}$ data were obtained by fitting at least three consecutive cycles of $\Delta P\left(t\right)$ with a sinusoidal curve by means of the Levenberg–Marquardt algorithm provided by OriginPro. In the inset, the signal-to-noise ratio S/N is also shown as a function of $d_{M}$.

In Figure 4a, the pressure drops of DI water are plotted versus *t* for one actuation cycle at 1 Hz, and for three different amplitudes of the imposed displacement. We estimate that the Womersley number $Wo=h\sqrt{\omega /\nu }\approx 0.1$ at such *f*. The phase angle $\phi_{\exp.}$ is approximately 90 degrees, which is expected for Newtonian fluids.

**Figure 5:**
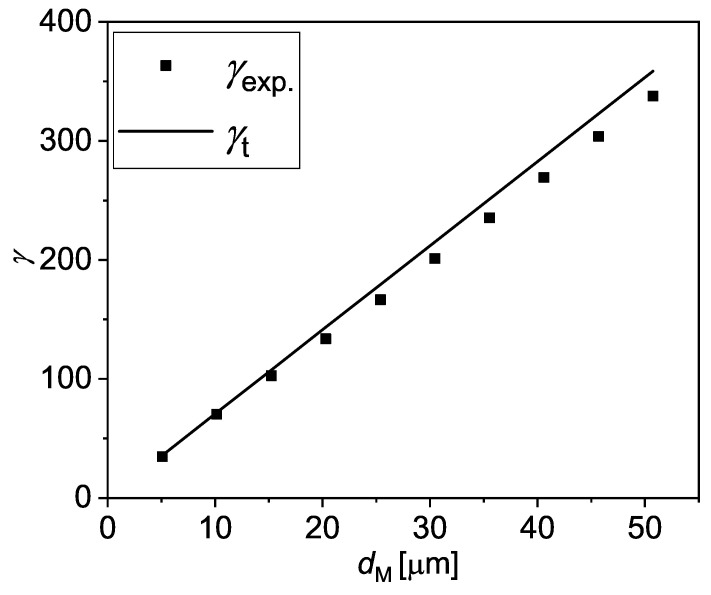
The theoretical ($\gamma_{t}$) and the experimental ($\gamma_{\exp.}$) strains at *f* = 1 Hz plotted versus $d_{M}$.

The measured $\Delta P_{M}$ and $\phi_{\exp.}$ are compared with the theoretical prediction given by Morris and Forster [17]. They derived an approximate expression for the impedance $\tilde{Z_{N}}=\tilde{\Delta P}/\tilde{Q}$ of oscillatory flows through rectangular ducts as

(1) $${\tilde{Z}}_{N}=R_{N}+i\omega I_{N},$$

where the resistance

(2) $$R_{N}=\frac{3\mu L}{4wh^{3}\alpha^{4}}\left(1-\frac{192\alpha }{\pi^{5}K}\right),$$

with α=w/h, is given by the steady state flow prediction due to White [23], and the inertance

(3) $$I_{N}=\frac{\rho L}{\alpha h^{2}}$$

takes the flow inertia into account. In the case of α = 1, the simplified expression of $\tilde{Z}$ obtained from Equations (1)–(3), resulted in being very close to the exact form obtained by solving the Navier–Stokes equations for oscillatory flow through straight ducts up to Wo≈10${}^{2}$. Based on such model, the theoretical $\Delta P_{M,t}$ is here computed as $\Delta P_{M,t}=\left|{\tilde{Z}}_{N}\right|Q_{M}$, where $Q_{M}=hwA_{\exp.}2\pi f$, $A_{\exp.}=kd_{M}$, and *k* is a fitting parameter. As shown in Figure 4a, the best fit of the $\Delta P_{M}$ data at 1 Hz is obtained for *k* = 166.3, while $\phi_{\exp.}$ resulted to be slightly below the theoretical $\phi_{N}=\arg\left(\tilde{\Delta P}\right)$. The inset of the Figure also shows the signal to noise ratio S/N, which is computed as

(4) $$S/N=\frac{\left|\Delta P_{M}\right|}{\sigma },$$

where σ is the standard deviation of the residual of the fitting of the ΔP versus *t* data with a simple sinusoidal wave. We obtain 10≤S/N≤60 for the data shown here, which demonstrates that the measured signal is always well above the sensitivity of the pressure sensors. Additionally, we also estimate an experimental amplitude of the fluid strain as $\gamma_{\exp.}=2A_{\exp.}/h$ and compare it with the theoretical one, $\gamma_{t}=2A_{t}/h$ in Figure 5. $A_{t}$ is hereby calculated assuming that the portion of the membrane covering the fluid displaces in a piston-like manner, i.e., $A_{t}=\pi d_{M}D_{c}^{2}/4hw$. We obtain that $\gamma_{\exp.}$ is very close to $\gamma_{t}$.

### 3.2. The Frequency Dependence of Pressure Drop Measurements Is Not Captured by the Newtonian Prediction

**Figure 6:**
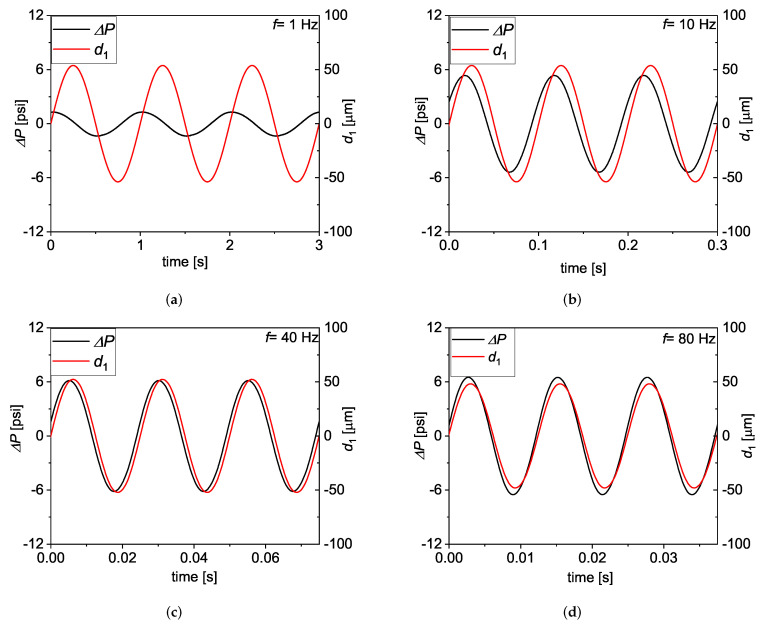
The measured $\Delta P\left(t\right)$ at $d_{M}$ = 50.75 μm, plotted versus *t* together with the corresponding $d_{1}\left(t\right)$ at *f* = 1 Hz (**a**), 10 Hz (**b**), 40 Hz (**c**), and 80 Hz (**d**).

**Figure 7:**
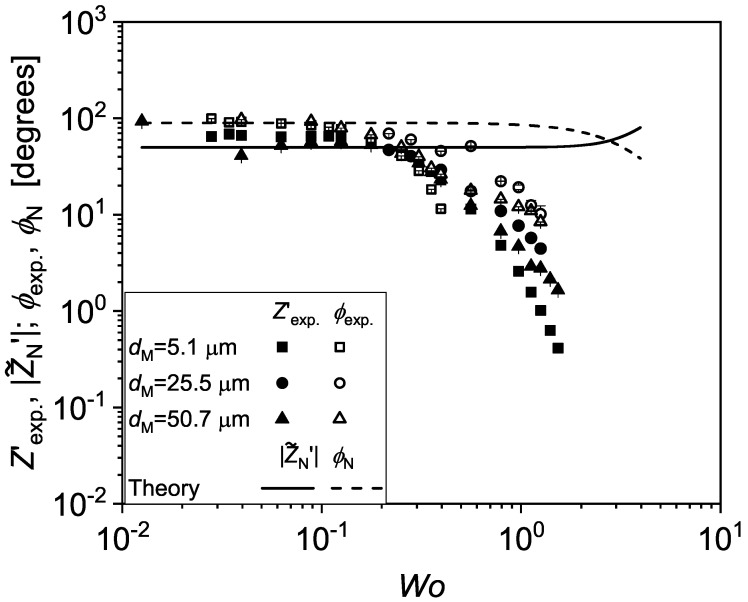
The experimentally measured non-dimensionalised compliance, $Z_{\exp.}^{\prime }=Z_{\exp.}4wh^{3}/\left(\mu L\right)$, and the phase $\phi_{\exp.}$ between $\Delta P\left(t\right)$ and $d_{1}\left(t\right)$ are plotted versus the Womersley number *Wo*. The continuous and dashed lines represent the non-dimensionalised modulus of the complex impedance, $|{\tilde{Z}}_{N}^{\prime }|$, and the phase $\phi_{N}$ obtained from the theoretical prediction for Newtonian fluids (See Equations (1)–(3)).

Having demonstrated that the measurements at low frequency closely resemble the Newtonian prediction, we now turn to the *f* dependence of the pressure drop. The ΔP versus *t* data are plotted in Figure 6, together with the corresponding $d_{1}\left(t\right)$ of the A1 actuator as measured by the strain gauge sensor, for 1 Hz≤f≤ 80 Hz and $d_{M}=50.7\;\mu$m. While the measured ΔP retains a sinusoidal form throughout the entire range of imposed *f*, it is interesting to notice that its amplitude $\Delta P_{M}$ levels up for f ≥ 10 Hz, while $\phi_{\exp.}$ tends to reduce as *f* is increased. A critical comparison of the experimental data with predictions derived from Newtonian theory is helpful to better understand the frequency response of the system under study. In Figure 7, the non-dimensionalised impedance, $Z_{\exp.}^{\prime }=Z_{\exp.}4\alpha h^{4}/\left(\mu L\right)$ where $Z_{\exp.}=\Delta P_{M}/Q_{M}$, and $\phi_{\exp.}$ are plotted versus Wo, and compared to the analytical predictions given by Equations (1)–(3), over a range of 0.05 Hz ≤f≤ 80 Hz, corresponding to 0.01 ≤Wo≤ 1.5. The experimental data fall below the analytical predictions at Wo≥ 0.2, which corresponds to *f* = 3 Hz, approximately.

### 3.3. Membrane Compliance Accounts for the Observed Frequency Dependence of Pressure Drop

In order to model the observed dependence of $\Delta P_{M}$ and $\phi_{\exp.}$ on *f*, we take a similar approach to that of Vedel et al. [19]. Therefore, we hypothesize that the pressure field generated by the water flow deforms the actuator membranes, so that a dead volume $V_{d}\left(t\right)$ is formed. At a first approximation, we assume that a linear relationship exists between $V_{d}\left(t\right)$ and the measured pressure drop across the microchannel, that is,

(5) $$V_{d}\left(t\right)=C\Delta P\left(t\right),$$

where *C* is a characteristic compliance. Therefore, the volume occupied by the fluid in the chamber 1, $V_{1,\mathrm{real}}$, can be written as

(6) $$V_{1,\mathrm{real}}\left(t\right)=\frac{\pi D_{c}^{2}}{4}\left[H-d_{1}\left(t\right)\right]+C\Delta P\left(t\right).$$

From Equation (6), we obtain a relationship between $Q_{\mathrm{real}}\left(t\right)=-\frac{dV_{\mathrm{real}}}{dt}$ and $Q\left(t\right)=\frac{\pi D_{c}^{2}}{4}\frac{d\left[d_{1}\left(t\right)\right]}{dt}$. In the complex domain,

(7) $${\tilde{Q}}_{\mathrm{real}}=\tilde{Q}-i\omega C\tilde{\Delta P}.$$

Because ${\tilde{Q}}_{\mathrm{real}}=\tilde{\Delta P}/{\tilde{Z}}_{N}$, the new relationship between $\tilde{\Delta P}$ and $\tilde{Q}$ becomes

(8) $$\tilde{\Delta P}=\frac{\tilde{Q}}{\frac{1}{{\tilde{Z}}_{N}}+i\omega C},$$

and a real impedance ${\tilde{Z}}_{\mathrm{real}}$ is then defined as

(9) $${\tilde{Z}}_{\mathrm{real}}=\frac{\tilde{\Delta P}}{\tilde{Q}}=\frac{{\tilde{Z}}_{N}}{{\tilde{Z}}_{N}+i\omega C{\tilde{Z}}_{N}}.$$

**Figure 8:**
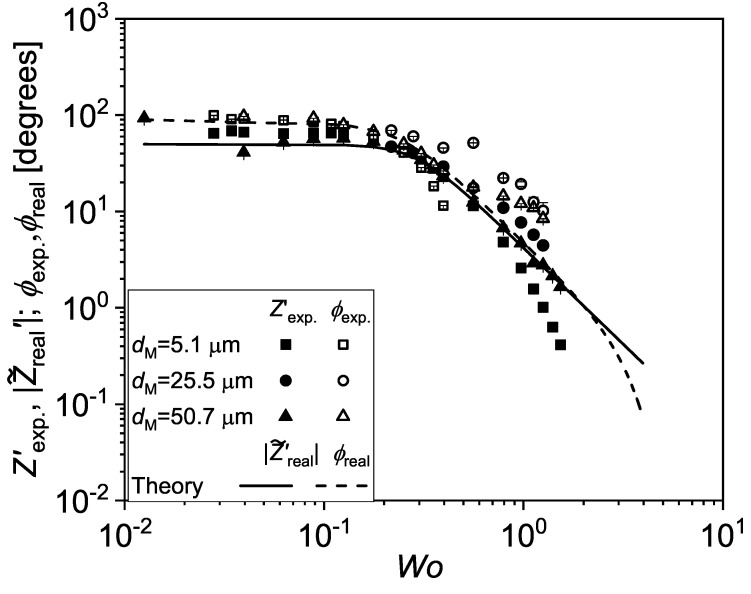
The experimental data are here compared with the prediction for the real impedance, ${\tilde{Z}}_{\mathrm{real}}$, obtained from Equation (9). The data and the theoretical predictions are non-dimensionalised in the same way as in Figure 7.

In Figure 8, $Z_{\exp.}$ and $\phi_{\exp.}$ are compared to the prediction from Equation (9), where *C* is the only fitting parameter. The best fit is obtained for $C\approx 0.8\times 10^{-14}$ m${}^{3}$/Pa, which is about one order of magnitude smaller than the value obtained by Vedel et al. ($C\approx 2\times 10^{-13}$ m${}^{3}/$Pa) for a similar system made with a more deformable rubber membrane. The model reproduces well the measured decay of the impedance and of phase up to Wo≈1. At Wo≥1, we see that $Z_{\exp.}^{\prime }<\left|{\tilde{Z}}_{\mathrm{real}}^{\prime }\right|$, which is most likely due to the onset of inertial effects.

## 4. Conclusions

We have tested a “Rheo-chip” prototypical rheometer for characterising oscillatory flow of model fluids over a range of frequencies 0.05 Hz ≤f≤ 80 Hz, corresponding to a range of the Womersley number 0.03 ≤Wo≤1.5, nominal strain 20 ≤γ≤ 350, and with sample consumption of less than 1 mL, by means of Newtonian flow. Compared to conventional (rotational) rheometers, such novel technique enables exploring a wider range of both frequency and strain, with minimum inertial effects, while sample consumption is reduced and interfacial effects are also avoided. While the measured amplitude of the shear strain at 1 Hz, $\gamma_{\exp.}$, resulted in being very close to the nominal strain $\gamma_{t}$, the measured impedance $Z_{\exp.}$ was close to the Newtonian prediction only up to Wo≈ 0.2 (corresponding to f≈ 3 Hz), after which the experimental data fell below the theoretical curves. We attribute such results to the deformability of the membranes used to couple the microfluidic chip with the piezo actuators. By introducing a simple model which assumes a linear dependence between ΔP and the dead volume within the fluid chambers, the frequency response of both the amplitude of the pressure drop $\Delta P_{M}$ and of the phase angle could be modeled more efficiently.

We are aware of the fact that our simplified model is only a first-order approximation of the complex relationship between the imposed flow rate and the measured pressure response. A more realistic approach needs to predict how the pressure field modifies the profile of the membrane [20]. From this viewpoint, the use of Discrete Fluidic Modeling (DFM) [24,25] would sensibly improve the accuracy of the prediction. DFM would be particularly useful for modeling the behaviour of non-Newtonian fluids under microfluidic oscillatory flow. This is because the presence of a viscoelastic component of the stress tensor is expected to further complicate the relationship between the membrane profile and the pressure field, resulting in a highly non-trivial dependence of $\Delta P_{M}$ on *f* at a fixed γ. Additionally, it is well known that, for several non-Newtonian fluids, the shear stress versus time curve under the LAOS regime is much different from a sine wave [26], which introduces a further complication to the development of a reliable model of the “Rheo-chip” system.

The effects observed in this work for a Newtonian fluid constitute a limitation of the technology described here, and need to be taken into account in future works using a similar approach for characterising the frequency response of complex fluids in microfluidics.

## Data Availability

The data presented in this study are available on request from the corresponding author. The data are not publicly available due to privacy.

## List of Symbols

α	width/depth channel aspect ratio
ΔP	Pressure drop
$\Delta P_{M,t}$	Theoretical amplitude of the pressure drop
$\Delta P_{M}$	Amplitude of the pressure drop
γ	Strain
$\gamma_{\exp.}$	Experimental amplitude of fluid strain
$\gamma_{t}$	Theoretical amplitude of fluid strain
λ	Longest relaxation time
μ	Shear viscosity
ν	Kinematic viscosity
ω	Angular frequency
$\phi_{\exp.}$	Phase angle between the pressure drop and the A1 actuator displacement
$\phi_{\mathrm{real}}$	Real phase delay
$\phi_{N}$	Newtonian phase delay
ρ	Fluid density
σ	Standard deviation of the residual of the sinusoidal fitting of $\Delta P\left(t\right)$
∼	Complex quantity
$A_{\exp.}$	Effective amplitude of fluid displacement within the microchannel
$A_{t}$	Theoretical amplitude of fluid displacement
*C*	Membrane compliance
$d_{1}$	Displacement of Actuator 1
$d_{2}$	Displacement of Actuator 2
$D_{c}$	Diameter of fluid chamber
$D_{H}$	Hydraulic diameter
$d_{M}$	Membrane diameter
*h*	Channel depth
$I_{N}$	Newtonian inertance
*K*	$\Sigma_{n=1,3,5...}^{+\infty }\frac{\tanh\left(\frac{n\pi }{2\alpha_{i}}\right)}{n^{5}}$
*L*	Distance between pressure sensors
$L_{P}$	Distance between the pressure sensor and the fluid chamber
$P_{0}$	Gauge pressure measured by PS1 sensor
$P_{1}$	Gauge pressure measured by PS2 sensor
*Q*	Flow rate
$Q_{\mathrm{real}}$	Real flow rate
$Q_{M}$	Amplitude of the flow rate
*R*	Membrane radius
$R_{1}$	Plate radius
$R_{N}$	Newtonian resistance
*t*	Time
$V_{1,\mathrm{real}}$	Real fluid volume occupied in chamber 1
$V_{d}$	Dead volume of fluid
*w*	Channel width
Wo	Womersley number
$Z_{\exp.}^{\prime }$	Non-dimensionalised, experimentally measured impedance
$Z_{\mathrm{real}}^{\prime }$	Non-dimensionalised, real impedance
$Z_{\exp.}$	Experimentally measured impedance
$Z_{\mathrm{real}}$	Real flow impedance
$Z_{N}$	Newtonian impedance
*f*	Frequency
De	Deborah number
